# Spike Detection Based on Normalized Correlation with Automatic Template Generation

**DOI:** 10.3390/s140611049

**Published:** 2014-06-23

**Authors:** Wen-Jyi Hwang, Szu-Huai Wang, Ya-Tzu Hsu

**Affiliations:** Department of Computer Science and Information Engineering, National Taiwan Normal University, Taipei 116, Taiwan; E-Mails: a0919779123@gmail.com (S.-H.W.); coolivan3@outlook.com (Y.-T.H.)

**Keywords:** spike sorting, spike detection, brain machine interface

## Abstract

A novel feedback-based spike detection algorithm for noisy spike trains is presented in this paper. It uses the information extracted from the results of spike classification for the enhancement of spike detection. The algorithm performs template matching for spike detection by a normalized correlator. The detected spikes are then sorted by the OSortalgorithm. The mean of spikes of each cluster produced by the OSort algorithm is used as the template of the normalized correlator for subsequent detection. The automatic generation and updating of templates enhance the robustness of the spike detection to input trains with various spike waveforms and noise levels. Experimental results show that the proposed algorithm operating in conjunction with OSort is an efficient design for attaining high detection and classification accuracy for spike sorting.

## 1. Introduction

Spike sorting [1] is often desired for the design of a brain machine interface (BMI) [2]. It receives spike trains from extracellular recording systems. Each spike train is a mixture of the trains from neurons near the recording electrodes. Spike sorting is able to segregate the spike trains of individual neurons from this mixture. It usually involves detection and classification operations. Spike detection is the first step of the spike sorting. The goal of spike detection is to separate spikes from background noise. Extracellularly recorded signals are inevitably corrupted by noise from a number of sources, such as the recording hardware and electromagnetic interference. In the presence of large noise, successful spike detection is essential for subsequent accurate classification.

One way to perform the spike detection is based on the amplitude or energy of spike trains. A simple amplitude-based spike detection [3] involves only the computation of the absolute value of spike samples. A spike is detected when the absolute value of a spike sample is above a threshold. Another approach is based on the nonlinear energy operators (NEOs) [4]. Commonly used operators, such as the Teager energy operator (TEO) and its variants, have been found to be effective [5,6]. In addition to NEO, the block energy of input trains can be used for performing generalized likelihood ratio tests (GLRTs) [7] for spike detection. Moreover, the detection can also be based on the energy of coefficients in the domain of stationary wavelet transform (SWT) [8,9]. The energy-based methods are simple and efficient. However, although the energy-based algorithms can operate in conjunction with a number of automatic thresholding algorithms [4,6,10], the proper selection of threshold values for these algorithms may still be difficult when noise becomes large. Therefore, their performance may deteriorate rapidly as noise energy increases.

An alternative to the energy-based methods is to utilize the templates of spikes for detection. A typical technique using templates is based on a matched filter [5,11,12]. It operates by correlating known templates with the input spike trains to detect the presence of the templates. It can also be viewed as a correlator performing likelihood ratio detection (LRT) [7]. The matched filter has been widely used in the communication systems for noisy signal detection. A drawback of the matched filter is that its performance is dependent on the selection of templates. The miss and false alarm rates may arise when large mismatches exist between the templates and the actual spikes. Moreover, because the number of neurons producing spikes may also not be known in advance, another issue is the difficulty to determine the number of templates in the filter. Efforts have been made to adaptively generate templates for spike detection in [13]. The templates are obtained by the computation of median values of spikes having peak values above an automatically selected threshold. In the algorithm, spikes from different neurons may share the same template. In the cases that an input sequence comprises spikes from a large number of neurons, a single template may not be suited for the detection of all of the spikes.

A common feature of the existing detection approaches is that their operations are based solely on the noisy spike trains. A feedback scheme for detection, extracting information from the results of subsequent feature extraction and/or clustering operations, may further improve performance. This is because the results may reveal useful information, such as the estimated number of active neurons and the estimated mean value of spikes produced by each neuron. Many software or hardware implementations [14–16] of feature extraction are based on principal component analysis (PCA) [17] or its variants. The K-means or fuzzy C-means (FCM) [18] algorithms are also widely used for clustering. However, long offline training may be required by these methods. It may then be difficult to employ these methods in a feedback loop for online spike detection.

In light of the facts stated above, the goal of this paper is to present a novel feedback-based spike detection algorithm for noisy spike trains. The algorithm performs template matching for spike detection by a normalized correlator. Its templates are obtained from the OSort (Online Sorter) [19] algorithm, which is an effective unsupervised algorithm for spike classification. It does not require offline training for feature extraction and clustering. As compared with sorting systems, such as WaveClus [10] and KlustaKwik [14], the OSort algorithm is more computationally effective [20]. Therefore, the OSort algorithm is employed in the proposed feedback system for automatic template generation and updating.

The selection of the threshold for template matching can be facilitated by the incorporation of the normalized correlator. In the proposed method, an upper bound of the squared distance measure for pattern matching can be used to determine the threshold value. A simple rule is derived for this purpose. In addition, a fast algorithm is proposed to accelerate the speed for correlation computation. The fast algorithm is based on a post-correlation normalization scheme for reducing the computation complexities. With the additional implementation of energy-based pre-screening operations, the computation time of the proposed normalized correlator could be less than that of the basic matched filter.

In the initial stage of the proposed algorithm, because no spike sorting results are available for estimation, the detection is then based on the GLRT using block energy [7]. Based on the detected spikes, the OSort provides the estimated number of clusters in the input spike train, and the estimated mean value of spikes associated with each train. These estimations are then used to determine the number of templates and the generation of each template in the proposed normalized correlator for the subsequent detection.

Evaluations of the proposed algorithm are made over synthesized spike trains with various noise levels. The simulator developed in [21] is adopted to generate extracellular recordings. The experimental results show that the proposed algorithm can be effectively combined with the OSort for automatic template generation and spike detection. For spike trains with high noise levels, the proposed algorithm significantly outperforms other energy- or template-based detection techniques. It can be used as a robust alternative for applications demanding high detection accuracy for noisy spikes.

## 2. The Algorithm

### 2.1. Matched Filter

We start with the basic matched filter technique for spike sorting, which can be implemented by convolving the spike trains with the pre-stored templates. For the sake of simplicity, we assume the matched filter contains only one template. Let *x*[*m*] be the *m*-th sample of the input spike train. Let **x***_m_* = [*x*[*m*],*x*[*m*− 1], …,*x*[*m* − *N* + *1*]]*^T^* be the *m*-th segment of the spike train, where *N* is the length of a spike. The template for matched filtering contains also *N* elements, denoted by **t** = [*t*[1], …, *t*[*N* − 1]]*^T^*. The matched filter output at *m*, denoted by, *y*[*m*], is computed from the convolution:

(1) $$y[m]=\overset{N-1}{\underset{k=1}{\text{∑}}}x\left[m-k\right]t[k]={\text{x}}_{m}^{T}\text{t}$$

Note that the convolution is equivalent to the inner product of segment **x***_m_* and template **t**, which indicates the correlation between these two vectors. The segment **x***_m_* is detected as a spike when *y*[*m*] is larger than a pre-specified threshold *η*.

### 2.2. LRT and GLRT

Under the assumption that the template **t** is the waveform of a known spike and the input spike train is corrupted by additive zero-mean white Gaussian noise **n**, the matched filter is equivalent to the LRT. Given the observation **x***_m_*, the goal of the LRT is to determine which of the following two hypotheses (denoted by *H*_0_ and *H*_1_, respectively) is consistent with the observation:

$$\begin{array}{l} H_{0}:{\text{x}}_{m}=\text{n}\\ H_{1}:{\text{x}}_{m}=\text{t}+\text{n} \end{array}$$

The probability density function of n is 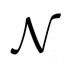
(0, Σ), where Σ is the covariance matrix of **n**. Because the Gaussian noise is white, Σ = σ^2^**I**, where *σ*^2^ is the noise variance, and **I** is the identity matrix. Therefore, the probability density function of **x***_m_* under the hypothesis *H*_0_, denoted by *P*(**x***_m_*/*H*_0_), is also 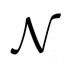
(0, Σ). Moreover, the probability density function under *H*_1_ is *P*(**x_m_**/*H*_1_) = 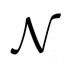
(**t**, Σ). That is,

(2) $$P\left({\text{x}}_{m}/H_{1}\right)=\frac{1}{\sqrt{2\pi \left|\text{∑}\right|}}e^{-\frac{1}{2}{\left({\text{x}}_{m}-\text{t}\right)}^{T}{\text{∑}}^{-1}\left({\text{x}}_{m}-\text{t}\right)}$$

where |Σ| is the determinant of the covariance matrix Σ. The optimal test is then given by [22]:

(3) xmT∑−1tH1><H0logP(H0)(c10−c00)P(H1)(c01−c11)≡γ

where c*_ij_* is the cost of deciding hypothesis *H_i_* when *H_j_* is in effect and *P*(*H_i_*) is the probability that **x***_m_* is in the mode *H_i_*. These quantities can be lumped into a single value γ. Because Σ = σ^2^**I**, LRT ${\text{x}}_{m}^{T}\;{\text{∑}}^{-1}\text{t}$ in Equation (3) is equivalent to the correlation operation ${\text{x}}_{m}^{T}\text{t}$ in Equation (1) with the threshold *η* = *σ*^2^*γ*.

Prior knowledge of spike waveforms is beneficial for detection based on matched filters. In practice, this information may not be fully available. One approach to solve this problem is to estimate **t** from **x***_m_*. Based on the assumption that **x***_m_* is in the mode *H*_1_, the maximum likelihood (ML) estimate of **t**, denoted by **tˆ**, selects **t**, maximizing *P*(**x***_m_*/H_1_) in Equation (2). This happens when **tˆ** = **x***_m_*. That is,

(4) $$\hat{\text{t}}=x_{\text{m}}=\arg\underset{\text{t}}{\max}P\left({\text{x}}_{m}/H_{1}\right)$$

In this case, the hypothesis *H*_1_ is not dependent on the template **t**, and the LRT becomes a GLRT. It takes the form:

(5) $$\begin{array}{ccc} {\text{x}}_{m}^{T}\;{\text{∑}}^{-1}{\text{x}}_{m} & \begin{array}{c} H_{1}\\ >\\ <\\ H_{0} \end{array} & \gamma . \end{array}$$

For the sake of simplicity, uniform cost (*i.e.*, *c*_00_ = *c*_11_ = 0, and *c*_01_ = *c*_10_ = 1) is assumed. In addition, without loss of generality, we also assume that the probability of the occurrence of a silent segment is larger than the occurrence of a spike (*i.e.*, *P*(*H*_0_) > *P*(*H*_1_)). It can then be observed from Equation (3) that γ > 0. With Σ = σ^2^**I**, the GLRT is equivalent to comparing the block energy ${\text{x}}_{m}^{T}{\text{x}}_{m}$ with the threshold *η* = *σ^2^γ* > 0. That is,

(6) $$\begin{array}{ccc} {\text{x}}_{m}^{T}{\text{x}}_{m} & \begin{array}{c} H_{1}\\ >\\ <\\ H_{0} \end{array} & \sigma^{2}\gamma \equiv \eta . \end{array}$$

The GLRT therefore is a block energy detector. With zero-mean assumption, one way to estimate *σ* (and subsequently *σ*^2^)for **x***_m_* is based on [10]

(7) $$\sigma =\text{median}\{\frac{\left|x[k]\right|}{0.6745},k=m-M+1,\ldots ,m\}$$

where *M* is the length of the input samples for finding the running median values for the estimation of *σ*. In addition to *σ*^2^, the value of γ is required to determine the threshold value *η*.

### 2.3. Normalized Correlator

A drawback of the matched filter or LRT is that the proper selection of a threshold value *η* may be challenging. From Equation (3), we see that, without the prior knowledge of *P*(*H*_0_), *P*(*H*_1_) and *σ*^2^, the computation of optimal *η* may be difficult. An alternative approach for the threshold selection is to determine the *η* value from an upper bound of a mismatch measurement for template matching. An input block with the mismatch measurement below the upper bound is declared as a hit, where the upper bound can be pre-specified. A correlator designed in this way can be linked to the simple pattern matching techniques based solely on the squared distance measures.

The normalized correlator may be effective for the design, where the mismatch measurement is based on the squared distance between the normalized template and the normalized observed input block.

Define **x̄***_m_* and **t̄** as the normalized version of **x***_m_* and **t**, respectively. That is,

(8) $$\begin{array}{cc} {\bar{\text{x}}}_{m}=\frac{{\text{x}}_{m}}{\left\|{\text{x}}_{m}\right\|}, & \bar{\text{t}}=\frac{\text{t}}{\left\|\text{t}\right\|} \end{array}$$

An advantage of the normalized correlator is that the squared distance between the normalized observed sequence **x̄***_m_* and the normalized template **t̄**, *d*(**x̄***_m_*, **t̄**), is dependent only on their correlations. It can be easily seen that:

(9) $$d\left({\bar{\text{x}}}_{m},\bar{\text{t}}\right)=2-2{\bar{\text{x}}}_{m}^{T}\bar{\text{t}}$$

Moreover, because *d*(**x̄***_m_*, **t̄**) > 0,

(10) $${\bar{\text{x}}}_{m}^{T}\bar{\text{t}}\leq 1$$

Our normalized correlator is based on **x̄***_m_* and **t̄**. When ${\bar{\text{x}}}_{m}^{T}\bar{\text{t}}>\eta$, then **x***_m_* is detected as a spike. From Equation (10), it follows that:

(11) η≤1

In addition, when **x**_m_ is detected as a spike (*i.e.*, ${\bar{\text{x}}}_{m}^{T}\bar{\text{t}}>\eta$), from Equation (9), we see that:

(12) $$d\left({\bar{\text{x}}}_{m}\bar{\text{t}}\right)\leq 2\left(1-\eta \right)\equiv D$$

where *D* can be viewed as the upper bound of the squared distance for a hit. Therefore, the threshold value for correlation computation uniquely determines the upper bound of the squared distance for template matching (and *vice versa*). In addition, a larger *η* implies a smaller mismatch *d*(**x̄***_m_*,**t̄**). The upper bound of *η* is one, which is independent of the input spike trains.

From the facts that the upper bound of the squared distance for a detected spike is *D* = 2(1 − *η*), where *η* < 1, the normalized correlator has a simple guideline for the selection of threshold value *η*. When *η* = 1.0 is selected for detection, only the segments having full correlation with the template t are considered as spikes, and their squared distance with t is zero. When *η* = 0.5, all of the segments having half correlation (or above) with t are detected as spikes, and the upper bound of their squared distances is one. When *η* = 0, even the segments having no correlation with t are detected as the spikes, and the upper bound of their squared distances increases to two. In the presence of noise, it may be impractical to require the detected spikes to be the segments having full correlation (*i.e.*, *η* = 1.0). In our experiments, the requirement of 50% correlation (*i.e.*, *η* = 0.5) may be sufficient for the normalized correlator to attain a high detection hit rate, low miss rate and low false alarm rate, even for high noise levels.

The computational cost of a direct implementation of the normalized correlator may be high. Although the normalization of the template **t** can be computed in advance, the most computational demanding part of the normalized correlator is the computation of **x̄***_m_*. It involves the computation of ‖**x**_m_‖ and the normalization ${\bar{\text{x}}}_{m}=\frac{{\text{x}}_{m}}{\left\|{\text{x}}_{m}\right\|}$. Because **x***_m_* consists of *N* spike samples, the computation of ‖**x**_m_‖ needs *N* multiplications, *N*− 1 additions and one squared root operation. Moreover, normalization requires *N* divisions. Finally, the inner product of ${\bar{\text{x}}}_{m}^{T}\bar{\text{t}}$ needs *N* multiplications and *N* − 1 additions. In total, the basic implementation of the normalized correlator requires 2*N* multiplications, (2*N* − 2) additions, *N* divisions and one squared root operation.

To expedite the computation, a simple fast implementation of the normalized correlator based on fast energy computation and post-correlation normalization is proposed. The fast energy computation exploits the correlation between **x***_m_*_−1_ and **x***_m_*. Observe that:

(13) $${\left\|{\text{x}}_{\text{m}}\right\|}^{2}={\left\|{\text{x}}_{m-1}\right\|}^{2}-x^{2}\left[m-N\right]+x^{2}[m]$$

Because ‖**x**_m−1_‖^2^ is already available after the computation of ${\bar{\text{x}}}_{m-1}^{T}\bar{\text{t}}$ is completed, the computation of ‖**x**_m_‖ needs only two multiplications, two additions and one squared root operation.

The post-correlation normalization is based on the observation that:

(14) $${\bar{\text{x}}}_{m}^{T}\bar{\text{t}}=\frac{1}{\left\|{\text{x}}_{m}\right\|}{\text{x}}_{m}^{T}\bar{\text{t}}$$

Therefore, given **x***_m_* and ‖**x**_m_‖, by computing the normalization after the correlation ${\text{x}}_{m}^{T}\bar{\text{t}}$, only *N* multiplications, *N* − 1 additions and one division are required for the implementation of Equation (14). Only one division is needed (instead of *N* divisions for the basic implementation), because the inner product ${\text{x}}_{m}^{T}\bar{\text{t}}$ produces only a scalar. In total, the fast implementation of the normalized correlator needs only *N* + 2 multiplications, *N* + 1 additions, one division and one squared root operation.

Table 1 compares the computational complexities of various template matching methods for computing one correlation between two vectors with dimension *N*. It can be observed from the table that the fast normalized correlator implementation needs only around 50% of additions and multiplications, as compared with its basic implementation counterpart. In addition, the number of divisions is reduced significantly from *N* to one.

**Figure 1.**
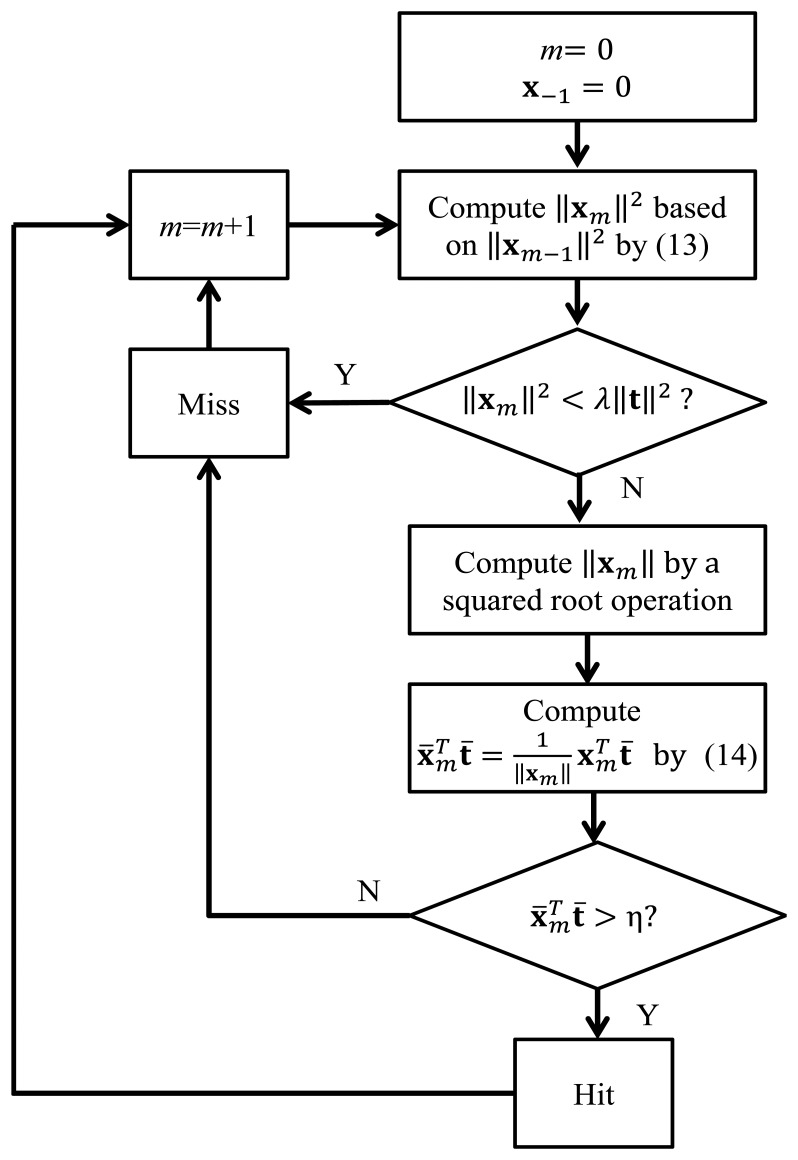
The operations of the fast normalized correlation computation with post-correlation normalization and energy-based pre-screening.

We can also see from Table 1 that the computational complexities of the fast normalized correlator implementation are only slightly higher than those of the matched filter without normalization. The computational complexities of the fast implementation can be further reduced by performing an energy-based pre-screening operation before the correlation computation. The pre-screening operation compares the energy ‖**x***_m_*‖^2^ with ‖**t**‖^2^. If ‖**x***_m_*‖^2^ < λ‖**t**‖^2^, where 0 < λ < 1, then ‖**x***_m_*‖^2^ may not be a spike, due to small energy. This may effectively reduce the number of correlation computations. By further incorporating the energy-based pre-screening operation, the proposed fast normalized correlator may have a computational time lower than that of the basic matched filter without normalization. Let *p* (0 < *p* < 1) be the probability that ‖**x***_m_*‖^2^ < λ‖**t**‖^2^ is true. As shown in Table 1, the average number of additions, multiplications and divisions are *p*(*N* − 1) + 2, *pN* + 2 and *p*, respectively. A flowchart detailing the operations of the fast normalized correlation computation with post-correlation normalization and energy-based pre-screening is shown in Figure 1.

Another practical issue for normalized correlation arises when correlation ${\bar{\text{x}}}_{m}^{T}\bar{\text{t}}$ varies slowly for successive *m* values. In this case, it may be possible that ${\bar{\text{x}}}_{m}^{T}\bar{\text{t}}>\eta$ for all *m* values in an interval 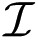 of integers. To avoid possible false alarms, for all of the blocks **x***_m_* with *m* values in 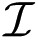, only the blocks having local maximum correlation values are considered as spikes.

### 2.4. OSort Algorithm

The OSort algorithm is an unsupervised template-based clustering algorithm for spike sorting. It does not require feature extraction, and the number of clusters is automatically determined by the algorithm. All of the detected spikes for clustering are aligned based on the maximum slope values of the spike waveforms [1].

Let **s** be the current detected spike to be classified. Let 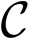
*_i_*, *i* = 1, …,*c*, be the current clusters produced by the OSort algorithm, where *c* is the number of clusters. Let **t***_i_*,*i* = 1, …,*c*, be the mean of the spikes belonging to 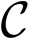
*_i_*. That is, **t***_i_* is the center of 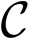
*_i_*. The OSort algorithm operates by first computing *d_i_* = *d*(**s**, **t***_i_*) for *i* = 1, …,*t.* It then finds i* = arg min*_i_ d_i_*. If the minimum distance d_i__*_ is less than a pre-specified threshold *τ*_1_, then the detected spike **s** is assigned to 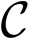
_i__*_. The corresponding mean **t**_i__*_ is then updated. Otherwise, a new cluster 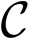
*_c_*_+1_ is created, and s is assigned to 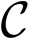
*_c_*_+1_. In this case, *c* is incremented by one.

**Figure 2.**
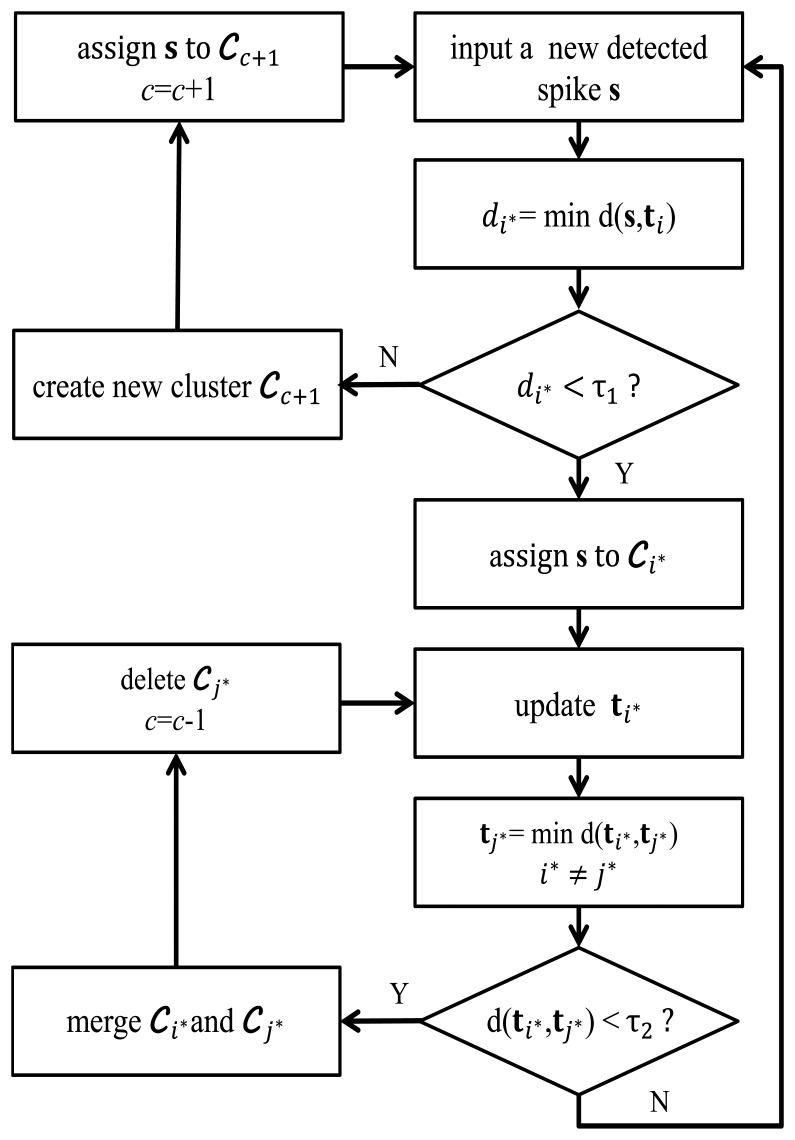
The flowchart of OSortoperations.

After the mean **t**_i__*_ is updated, the distance between **t**_i__*_ and **t***_j_*, *j* ≠ i* will be computed. Let j* = arg min*_j,j_*_≠__i__*_d(**t**_i__*_,**t***_j_*). We then compare *d*(**t**_i__*_,**t***_j_*) with another threshold value *τ*_2_. Both 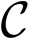
_i__*_ and *
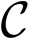
_j_*_*_ will be merged when *d*(**t**_i__*_,**t***_j_*) < *τ*_2_. In this case, *c* is decremented by one. A flowchart summarizing the operations of the OSort algorithm is shown in Figure 2.

After an interval of *T*_1_ seconds, the current **t***_i_*, *i* =1, …,*c*, are then used as the templates of the normalized correlator for spike detection. The interval length *T*_1_ can also be pre-specified. It may be beneficial to perform some simple validation operations before using **t***_i_*, *i* = 1, …, *c*, as templates. For example, when the number of spikes in a cluster 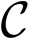
*_j_* is significantly smaller than that of other clusters, it is likely that the spikes in the 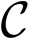
*_j_* are actually the noises. Therefore, we may remove **t***_j_* from the list of templates. Another simple criterion is to check the difference in time between the maximum and minimum samples of the **t***_j_*. If the difference in time is large and is close to the length of the spike, **t***_j_* may also be an average value of noises and can be removed.

### 2.5. Spike Detection/Sorting Based on GLRT, Normalized Correlator and OSort Algorithms

**Figure 3.**
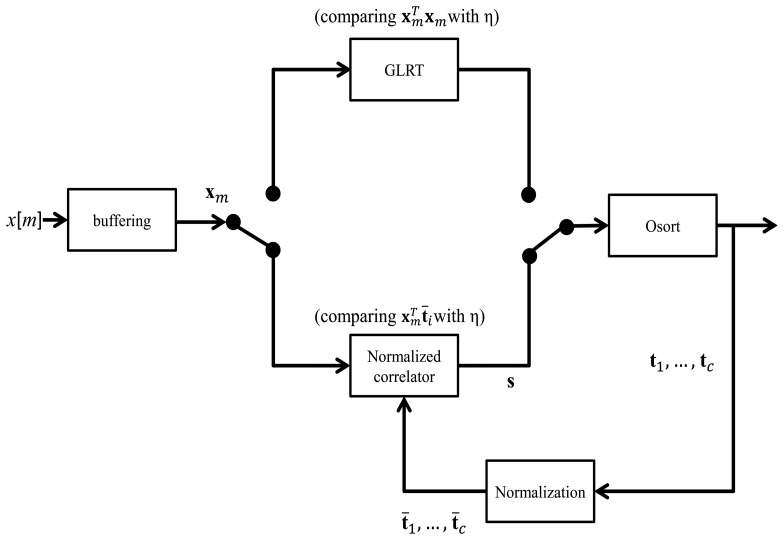
The block diagram of the spike detection/sorting system based on the generalized likelihood ratio test (GLRT), normalized correlator and OSort algorithms.

Figure 3 shows the block diagram of the spike detection/sorting system based on GLRT, normalized correlator and OSort algorithms. Both the GLRT and normalized correlator are used for spike detection, and OSort is used for spike classification. The system features automatic template generation. It is a feedback system, where the templates produced by OSort are used for the spike detection.

At the initial stage of the system, no templates are available for spike detection. Therefore, at this stage, the noncoherent block energy detector based on Equation (5) is adopted for detection. The threshold η in the initial stage is automatically determined by Equations (6) and (7). The detected spikes are then processed immediately by the OSort algorithm for classification and template generation. After a time interval of *T*_1_ seconds, the cluster centers **t***_i_*, *i* = 1, …, *c*, produced by OSort are used as templates for the subsequent spike detection.

Based on these templates, the normalized correlator computes ${\bar{\text{x}}}_{m}^{T}{\bar{\text{t}}}_{i},i=1,\ldots ,c$. The input block is detected as a spike when any of the *c* normalized correlation exceed the threshold *η*. Because of the normalized correlation operations, the threshold value is bounded, as shown in Equation (11). In addition, it is related to the upper bound of the squared distance for a hit by Equation (12). To ensure a fixed quality for a hit, a fixed threshold *η* can be used. The cluster centers produced by OSort after the detection and classification of every *T*_2_ seconds can also be used for the updating of templates. Constant updating of templates may be beneficial for the tracking of variations of input signals over a long recording period.

## 3. Experimental Results

The experiments for evaluating the performance of the proposed algorithm are based on the simulator developed in [21] for producing extracellular recordings. The simulator gives access to ground-truth about spiking activity in the recording. It facilitates a quantitative assessment of algorithm performance, since the features of the spike trains are known *a priori*. Various sets of spike trains with different signal-to-noise (SNR) ratios have been created by the simulator for our experiments. Let $\sigma_{s}^{2}$ be the average power of a spike train without noise. The addition of the spike train with a noise having average power *σ*^2^ results in a noisy spike train with the noise level defined as $\text{SNR}=\text{10}\log\frac{\sigma_{s}^{2}}{\sigma^{2}}$. The default sampling rate for the experiments is 24,000 samples/s. The length of each spike in the spike trains is 2.67 ms. Therefore, each spike has 64 samples (*i.e.*, *N* = 64) for the default sampling rate.

We first measure the computation time of various template matching methods for spike detection. The computation time of a template matching method is defined as the total time required for the spike detection of a spike train. In the experiments, spike trains with different SNR levels (*i.e.*, −2 dB, 0 dB, 2 dB, 4 dB, 6 dB and 8 dB) are considered. The recording time of each spike train is 100 s. The number of active neurons producing spikes is two. All of the algorithms are implemented by **C** codes for the comparison. The software programs are running on a 3.4-GHz Intel I7 processor with 16 Gb main memory.

The experimental results show that the computation time of the matched filter and basic normalized correlator are 0.67 s and 1.58 s, respectively. The computation time of both methods are independent of SNR levels. In addition, the basic normalized correlator has a computation time higher than that of the matched filter. This is because the normalized correlator without fast implementation requires a larger number of arithmetic operations, as revealed in Table 1. These arithmetic operations can be significantly reduced by the fast energy computation shown in Equation (13) and post-correlation normalization in Equation (14). With the further aid of energy-based pre-screening, the computation time of the normalized correlator is lower than that of the matched filter.

In fact, with λ = 0.5 for energy-based pre-screening, the computation time of the fast normalized correlator is 0.44 s, 0.40 s, 0.37 s, 0.28 s, 0.27 s and 0.26 s for SNR levels −2 dB, 0 dB, 2 dB, 4 dB, 6 dB and 8 dB, respectively. As compared with the matched filter, the proposed fast computation implementation is able to achieve up to a 61.19% reduction in computation time (*i.e.*, from 0.67 s to 0.26 s) for spike trains with high SNR = 8 dB. Higher SNR values are beneficial, because the silent segments are easier to identify (*i.e.*, *p* in Table 1 is smaller). However, even when SNR values are below zero, the fast implementation is still able to reduce the computation time. These results reveal the fact that the employment of a normalized correlator will not incur additional computation time for spike detection, as compared with its matched filter counterparts.

**Figure 4.**
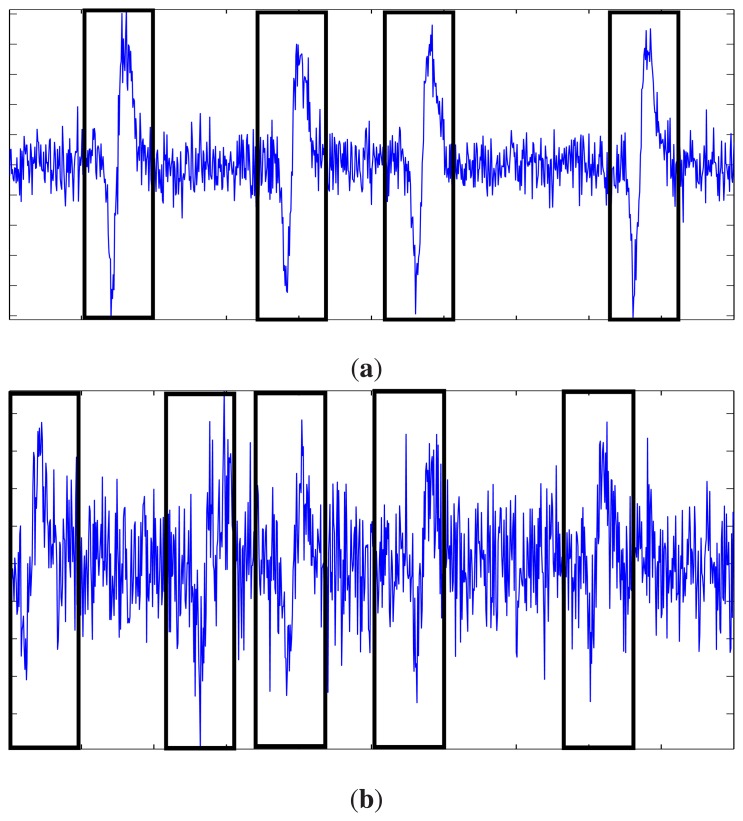
Samples of spike trains with various SNR levels. Spikes are marked with black rectangles. (**a**) SNR = 8 dB; (**b**) SNR = − 2 dB.

Spike detection could be a difficult problem when the SNR level of a spike train is low. Figure 4 shows samples of spike trains with SNR = 8 dB and −2 dB, respectively. Locations of spikes in spike trains are also marked in the figure. It can be observed from Figure 4 that it may be difficult to identify the spikes visually for low SNR levels, such as SNR = −2 dB. The proposed algorithm is helpful for the identification of the noisy spikes. We now consider the evaluation of the true positive (TP) rate and false alarm (FA) rate of the proposed spike detection algorithm. The TP rate is defined as the total number of detected spikes divided by the total number of spikes. The FA rate is defined as the total number of silent segments, which are detected as spikes divided by the total number of detected segments. The evaluation involves the measurement of TP and FA rates of spike trains with various SNR levels. In addition, for each spike train, the TP and FA rates of the initial stage and the second stage of the proposed algorithm are evaluated independently.

The detection at the first stage is based on the GLRT detection without a template. The selection of threshold *η* in the initial stage is based on Equations (6) and (7). That is, *η* = γ*σ*^2^, where *σ* is adaptively estimated by Equation (7). The estimation of γ may be difficult without the prior knowledge of *P*(*H*_0_) and *P*(*H*_1_) by Equation (5). One way to solve this problem is by first observing that *Nσ*^2^ is the average energy of the noise block without the presence of spikes. Therefore, an empirical approach to select γ in Equation (6) is γ = 1.2, *N* = 76.8. The approach ensures that a block is detected as a spike only when the average energy of the block is sufficiently larger than *Nσ*^2^.

The detection at the second stage is based on the templates obtained from the initial stage using OSort. The threshold values *τ*_1_ and *τ*_2_ for splitting and merging are computed in accordance with the method presented in [19], which is based on the variances of the detected spikes. The threshold for the normalized correlator is *η* = 0.7. In this experiment, the number of active neurons producing spikes is two. Table 2 shows the results of the evaluation, where the length of the first stage and the second stage are *T*_1_ = 2 s and *T*_2_ = 20 s, respectively.

Because the GLRT detection at the first stage of the proposed algorithm is based on a non-coherent energy test without template information, it may not be able to perform well when the SNR level becomes low. However, with the aid of templates, the detection at the subsequent stages are able to achieve a high TP rate and a low FA rate, even for SNR = −2 dB, as shown in Table 2. In fact, the TP and FR rates at the second stage are 82.39% and 0.71% when SNR = −2 dB, respectively. For other, higher SNR levels, the TP rates are above 88%, while FR rates are below 1% at the second stage. These results demonstrate the effectiveness of the proposed automatic template generation scheme.

Based on Table 2, we also see that the performance of the proposed algorithm at the second stage is robust against the FA rates at the first stage. As shown in the table, noisy spike trains with different SNR levels may produce different FA rates at the first stage. The FA rate grows from 5.12% to 20.25% when the SNR level reduces from 8 dB to −2 dB. Even with the FA rate of 20.25% at the first stage, the proposed algorithm is still able to achieve a TP rate of 82.39% and an FA rate of 0.71% at the second stage.

The detection performance at the first stage is inferior to that at the second stage. Therefore, it may be desirable to reduce the length of first stage *T*_1_. However, insufficient *T*_1_ may result in an insufficient number of spikes for template generation in OSort. This may degrade the performance at Stage 2. Table 3 reveals the performance at the second stage with *η* = 0.7 for various *T*_1_ values. It can be observed from Table 3 that the performance of the proposed algorithm at the second stage improves as *T*_1_ increases until it reaches 2 s. Afterward, the performance is not significantly enhanced by increasing *T*_1_. Therefore, we select *T*_1_ = 2 s as the lowest *T*_1_ for high performance at the second stage.

Next, we observe the performance at the second stage for different sampling rates for the spike trains with SNR = −2 dB. In addition to the default setting of 24,000 samples/s, two additional sampling rates, 12,000 samples/s and 48,000 samples/s, are also considered. The threshold value is *η* = 0.7. For a sampling rate of 24,000 samples/s, the TP rate and FA rate are 82.39% and 0.71%, respectively. When the sampling rate is lowered to 12,000 samples/s, the TP rate and FA rate are slightly degraded to 81.24% and 3.24%, respectively. The performance at the second stage is enhanced with a TP rate of 90.21% and an FA rate of 0.21% for a sampling rate of 48,000 samples/s. The proposed algorithm therefore has robust performance to the variations in sampling rates.

**Figure 5.**
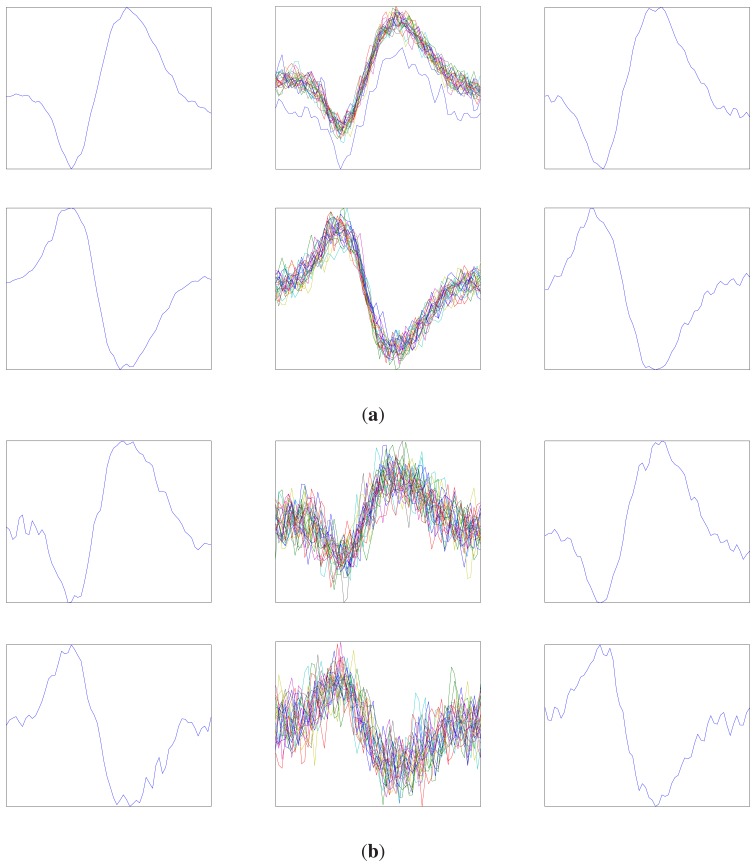
The average value of the noisy spikes produced by two active neurons (the first columns), the noisy spikes mapped to each cluster of OSort (the second column) and the templates obtained from OSort (the third column): (**a**) 8 dB; (**b**) −2 dB.

Figure 5 shows the average value of the noisy spikes produced by active neurons (the first column), the noisy spikes mapped to each cluster of OSort (the second column) and the templates obtained from OSort (the third column) for the SNR level of −2 dB and 8 dB at the first stage of the proposed algorithm. The time of the observation is *T*_1_ = 2 s. Under the energy-based detection of the GLRT at the first stage, we observe from Figure 5 that the center of each cluster provided by OSort still resembles the average value of the spikes produced by the corresponding neuron. Therefore, the centers of the clusters in the OSort algorithm are beneficial for enhancing the detection at subsequent stages.

In addition to having high detection performance, the proposed algorithm has an advantage of simple threshold selection for the normalized correlator. The threshold value *η* for the normalized correlator at the second stage need not to be adaptive. It is related to the upper bound of the squared distance *D* for a hit by Equation (12). Table 4 shows the detection performance of the proposed algorithm for various threshold values *η.* It can be observed from Table 4 that a larger *η* value is able to reduce the FA rate, because of the lowering of the upper bound *D* of the squared distance for a hit. Conversely, a lower *η* value increases the TP rate, even for low SNR levels. In our experiment, *η* = 0.7 achieves both high TP and low FA rates for all of the SNR levels under consideration.

In the following, we further elaborate on the selection of thresholds in the proposed algorithm. Recall that the FA rate is defined by the total number of silent segments that are detected as spikes divided by the total number of detected segments. The FA rate therefore indicates the percentage of the detected segments that are not spikes. The FA rate not only depends on the number of silent segments detected as spikes, but also the number of detected spike segments. Let:

(15) $$g({\text{x}}_{m})=\underset{1\leq i\leq c}{\max}\left({\bar{\text{x}}}_{m}^{T}{\bar{\text{t}}}_{i}\right)$$

That is, *g*(**x***_m_*) is the maximum value of the normalized correlation between a segment **x***_m_* and a template **t***_i_*. Let *f*_1_ be the distribution of *g*(**x***_m_*) for all silent segments in the noisy spike train and *f_2_* be the distribution for all spike segments. The dependency of FA on the threshold *η* can be observed from *f*_1_ and *f*_2_.

**Figure 6.**
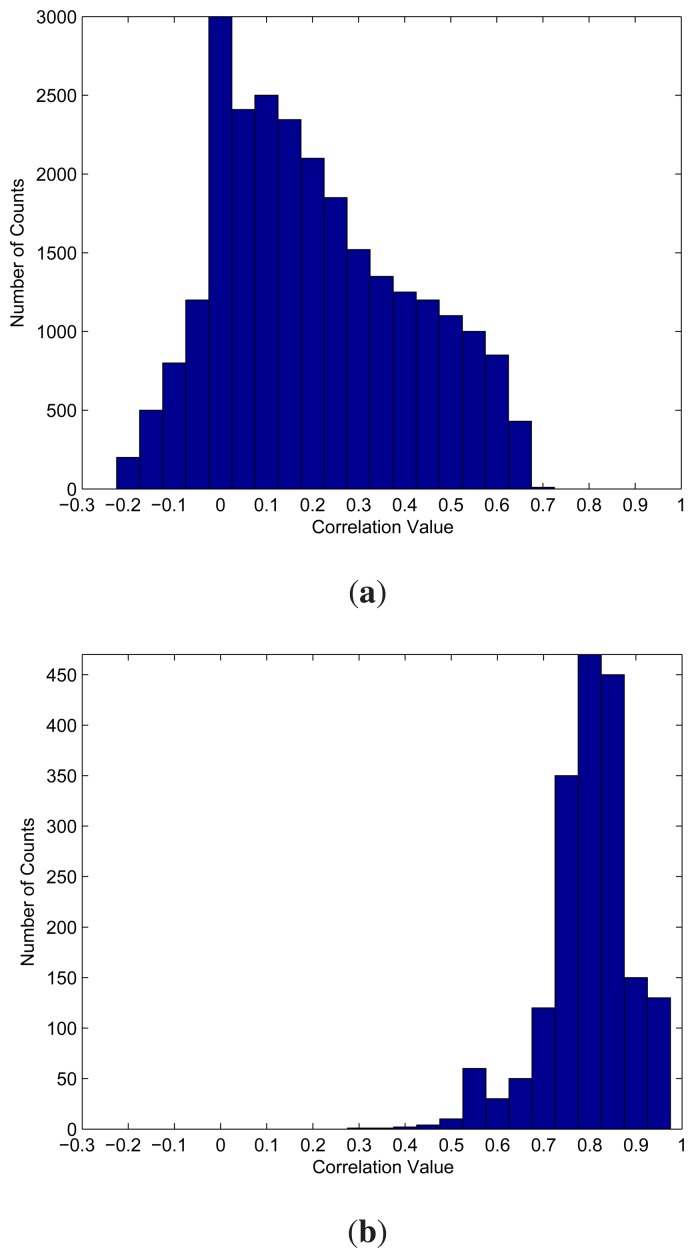
Distributions *f*_1_ and *f*_2_ for the spike train with SNR = − 2 dB. (**a**) distribution *f*_1_; (**b**) distribution *f_2_*.

Figure 6 shows *f*_1_ and *f*_2_ for the spike train with SNR =−2 dB used by the experiments for Table 4. Given a *η* > 0, let *F*_1_ (*η*) be the area above the *η* in *f*_1_. Because the normalized correlation should be less than one,

(16) $$F_{1}(\eta )=\int_{\eta }^{1}f_{1}(a)da$$

Therefore, *F*_1_(*η*) is the total number of detected segments that are not spikes. In addition, the area above the *η* in *f*_2_, denoted by *F*_2_(*η*), is the total number of detected spike segments. The corresponding FA rate can then be computed by:

(17) $$FA=\frac{F_{1}(\eta )}{F_{1}(\eta )+F_{2}(\eta )}$$

It is interesting to note that, because of the low correlation between noise and templates, when *η* is high (e.g., 0.7 or above), the *F*_1_(*η*) is small. On the other hand, because the templates are highly correlated with the spikes, *F*_2_ (*η*) is large for high *η.* As a result, *FA* is low for high *η*. As the *η* decreases, the *F*_2_(*η*) remains almost a constant, because only a very small fraction of spikes have a low correlation with the templates. The accumulation of this small fraction to *F*_2_(*η*) does not significantly increase the value. By contrast, the increases in *F*_1_(*η*) becomes relatively large. In particular, for *η* = 0.7 and 0.65, it can be observed from Figure 6 that *F*_1_(0.7) = 10 and *F*_1_(0.65) = 430. In addition, *F*_2_(0.7) = 1400 and F_2_(0.65) = 1450. Therefore, FA increases from 0.71% for *η* = 0.7 to 22.87% for *η* = 0.65. Although FA rapidly increases as *η* decreases from 0.7 to 0.65, the increases in *F*_1_ relative to the total number of silent segments (*i.e.*, *F*_1_(−1)) is actually quite small. Actually, *F*_1_ will grow significantly only when *η* decreases below 0.6, which is not recommended as the threshold value.

It can also be observed from the distribution *f*_1_ in Figure 6 that most of the *g*(**x***_m_*) is below 0.7 when **x***_m_* is a silent segment. Moreover, most of the *g*(**x***_m_*) is above 0.7 when **x***_m_* is a spike segment. Similar observations also hold for many of the other spike trains produced by different numbers of active neurons with various SNR levels. Table 5 shows the performance of the proposed algorithm for the spike trains produced by various numbers of active neurons with SNR levels = − 2 dB and 8 dB. Different threshold values are considered in the experiments. It can be observed from the table that *η* = 0.7 performs well for most of the cases.

To further evaluate the proposed algorithm, Table 6 compares the TP and FA rates of the proposed algorithm with those of the existing approaches for the spike trains with various SNR values. The number of active neurons is two in the experiments. The length of each spike train is 20 s. The proposed algorithm requires an additional stage (*i.e.*, first stage) for generating templates. The performance of the proposed algorithm is measured at the second stage after the automatic generation of templates. For the matched filter technique [11], the spikes are assumed to be pre-known for the generation of the templates. We can see from Table 6 that the proposed algorithm outperforms others, because of the utilization of adaptive templates and the simplicity for threshold selection. Although the matched filter uses accurate spikes as templates, its performance is still inferior to the proposed algorithm. This is because the selection of threshold values is more difficult for the matched filter. It will be dependent on the energy of spikes and noises. A single threshold may not be well-suited for all of the noisy spikes with different SNR levels. Although the energy-based techniques, nonlinear energy operators (NEOs) [4] and stationary wavelet transform (SWT) [8], perform well for high SNR values, their performance deteriorates rapidly as the noise energy grows. In particular, when SNR = −3 dB, the FA rate of the proposed algorithm is only 1%. By contrast, the FA rates of the the energy-based techniques are above 50% when SNR = −3 dB. The simple detection algorithm based on the absolute value of spike samples with automatic threshold value determination [10] has low FA rates. However, its TP rates are also low, as compared with other methods. Therefore, the proposed algorithm is effective for spike trains with large noise levels.

Tables 7 and 8 show the performance of OSort-based spike sorting systems using the proposed spike detection algorithm for spike trains generated by two and three active neurons with various SNR levels, respectively. The length of each spike train is 20 s. In the experiments, the number of undetected spikes and the number of detected, but misclassified, spikes are measured. The misclassified spikes are defined as the spikes that are assigned to the clusters by OSort that are different from their ground-truth. It can be observed from Tables 7 and 8 that the performance of the system is robust against the noises. The number of undetected spikes remains low, even for lower SNR levels. The cases of three active neurons may produce a larger number of clusters compared with their counterparts with two active neurons. Therefore, they are more likely to introduce higher classification error rates. Nevertheless, even for a low SNR level of −2 dB, more than 72% of spikes can still be detected and correctly classified by the proposed system for the cases of three active neurons. All of these facts show the effectiveness of the proposed algorithm.

## 4. Concluding Remarks

The proposed algorithm, combining normalized correlator with the OSort algorithm, has been found to be effective for spike detection. As compared with a traditional matched filter for template matching, the proposed normalized correlator with fast energy computation, post energy normalization and pre-screening has lower computation time. As compared with the basic normalized correlator approach, the reduction in computation time is up to 83.54% (from 1.58 s to 0.26 s). The reduction in computation time compared with the matched filter is 61.19% (from 0.67 s to 0.26 s). Therefore, the proposed algorithm is beneficial for the implementation of real-time detection. For a sampling rate of 24,000 samples/s, a two-second duration at the first stage would suffice to achieve high performance spike detection at the second stage. A single threshold value for the normalized correlator could be effective for a wide range of SNR levels. Because of the effectiveness of the automatic template generation, the proposed algorithm has a high TP rate and a low FA rate, even for low SNR levels. In particular, when SNR = −2 dB, the proposed algorithm is able to achieve a 82.39% TP rate and a 0.41% FA rate. It outperforms other energy-based or template-based techniques for spike detection. The spike sorting system based on the proposed detection algorithm also attains high classification accuracy, even at low SNR levels. The classification performance may be slightly degraded when there are more than two active neurons. However, more than 72% of the spikes can still be detected and correctly classified by the proposed system for the cases of three active neurons. Accurate detection and classification for a large number of neurons is an interesting future issue to be considered.

## Figures and Tables

**Table 1.** Computational complexities of various template matching methods for computing one correlation between two vectors with dimension *N*.

	**Addition**	**Multiplication**	**Division**	**Squared Root**
Matched				
Filter	*N* −1	*N*	0	0
Basic Normalized				
Correlator	2*N* −2	2*N*	*N*	1
Fast Normalized				
Correlator (No Pre-Screening)	*N* +1	*N* +2	1	1
Fast Normalized				
Correlator (Pre-Screening)	*p* × (*N* − 1) + 2	*p* × *N* +2	*p*	1

**Table 2.** The TP and FR rates of the proposed algorithm for spike trains with various SNR levels.

**SNR (dB)**		−**2**	**0**	**2**	**4**	**6**	**8**
First	TP Rate	83.37%	87.75%	86.64%	87.03%	90.18%	89.42%
	
Stage	FA Rate	20.25%	15.17%	9.45%	9.46%	7.26%	5.12%

Second	TP Rate	82.39%	88.22%	89.47%	90.74%	92.92%	93.04%
	
Stage	FA Rate	0.71%	0.59%	1.06%	0.91%	0.55%	0.36%

**Table 3.** The TP and FR rates at the second stage of the proposed algorithm for various *T*_1_ values.

***T*_1_ (s)**	**0.5**	**1.0**	**1.5**	**2.0**	**2.5**	**3.0**
TP Rate	48.85%	82.12%	83.71%	82.39%	82.24%	82.46%
FA Rate	67.47%	15.35%	5.49%	0.71%	0.72%	0.67%

**Table 4.** The TP and FR rates of the the second stage of the proposed algorithm based on various threshold values *η.*

**SNR (dB)**		−**2**	**0**	**2**	**4**	**6**	**8**
*η* = 0.8	TP Rate	76.47%	84.63%	86.47%	87.34%	89.18%	88.10%
	
	FA Rate	0.15%	0.52%	0.52%	0.90%	0.23%	0.12%

*η* = 0.75	TP Rate	79.41%	90.66%	88.37%	90.65%	90.09%	91.32%
	
	FA Rate	0.22%	1.92%	0.43%	0.47%	0.26%	0.45%

*η* = 0.7	TP Rate	82.39%	88.22%	89.47%	90.74%	92.92%	93.04%
	
	FA Rate	0.71%	0.59%	1.06%	0.91%	0.55%	0.36%

*η* = 0.65	TP Rate	90.51%	92.18%	95.67%	96.34%	95.03%	94.33%
	
	FA Rate	22.87%	40.26%	20.51%	14.28%	18.72%	20.54%

**Table 5.** The TP and FR rates at the second stage of the proposed algorithm for spike trains produced by a different number of neurons c with various SNR levels.

		**SNR** = **8 dB**			**SNR** = −**2 dB**		
	**Threshold *η***	**0.7**	**0.65**	**0.6**	**0.7**	**0.65**	**0.6**
*c*=2	TP Rate	93.04%	94.33%	95.22%	82.39%	90.51%	94.00%
	FR Rate	0.36%	20.54%	28.15%	0.71%	22.87%	35.71%

*c*=3	TP Rate	89.32%	90.33%	93.47%	82.08%	89.34%	93.41%
	FR Rate	3.26%	20.56%	31.75%	4.22%	21.65%	38.35%

**Table 6.** The TP and FR rates of various spike detection algorithms for spike trains with various SNR levels. NEO, nonlinear energy operator; SWT, stationary wavelet transform.

**SNR (dB)**		−**3**	−**2**	**0**	**1**	**8**	**10**
Proposed	TP Rate	82.71%	82.39%	88.22%	90.04%	93.04%	93.64%
	
Algorithm	FA Rate	1.06%	0.41%	0.59%	0.92%	0.36%	0.40%

NEO	TP Rate	80.53%	82.05%	83.47%	87.21%	92.24%	93.10%
	
Algorithm [4]	FA Rate	57.87%	56.16%	39.62%	22.49%	7.75%	3.57%

SWT	TP Rate	86.66%	87.38%	91.37%	92.43%	94.26%	94.82%
	
Algorithm [8]	FA Rate	82.43%	81.80%	79.00%	79.36%	19.00%	6.77%

Absolute	TP Rate	22.22%	51.08%	54.65%	67.81%	81.74%	90.90%
	
Value [10]	FA Rate	3.70%	0.84%	1.52%	1.25%	12.44%	17.96%

Matched	TP Rate	80.31%	80.83%	82.20%	82.90%	86.32%	89.65%
	
Filter [11]	FA Rate	8.92%	7.69%	4.62%	3.02%	2.94%	2.80%

**Table 7.** The performance of OSort-based spike sorting systems using the proposed spike detection algorithm for spike trains generated by two active neurons with various SNR levels.

**SNR**	**Total Number of Spikes**	**Number of Spikes not Detected**	**Number of Spikes Detected & Misclassified**	**Number of Spikes Detected & Correctly Classified**
−2	2,378	406 (17.07%)	10 (0.42%)	1,962 (82.51%)
0	2,308	260 (11.27%)	11 (0.48%)	2,035 (88.17%)
2	2,375	240 (10.10%)	10 (0.42%)	2,115 (89.05%)
4	2,387	197 (8.25%)	10 (0.42%)	2,180 (91.32%)
6	2,359	194 (8.22%)	9 (0.38%)	2,156 (91.38%)
8	2,332	165 (7.08%)	7 (0.30%)	2,160 (92.62%)

**Table 8.** The performance of OSort-based spike sorting systems using the proposed spike detection algorithm for spike trains generated by three active neurons with various SNR levels.

**SNR**	**Total Number of Spikes**	**Number of Spikes not Detected**	**Number of Spikes Detected & Misclassified**	**Number of Spikes Detected & Correctly Classified**
−2	2,014	354 (17.57%)	206 (10.23%)	1,454 (72.19%)
0	1,990	327 (16.43%)	194 (9.75%)	1,469 (73.81%)
2	1,983	217 (10.94%)	153 (7.71%)	1,613 (81.34%)
4	1,988	227 (11.42%)	81 (4.07%)	1,680 (84.51%)
6	2,008	219 (10.91%)	166 (8.27%)	1,623 (80.83%)
8	1,987	179 (9.00%)	126 (6.34%)	1,682 (84.65%)
